# Butterfly Pea Flower as a Novel Ingredient to Produce Antioxidant-Enriched Yellow Pea-Based Breakfast Cereals

**DOI:** 10.3390/foods11213447

**Published:** 2022-10-30

**Authors:** Ravinder Singh, Cheng-Chia Yu, Guan-Wei Chen, Ching-Hsueh Chen, Nasibeh Y. Sinaki, Jenshinn Lin, Filiz Koksel

**Affiliations:** 1 Department of Food and Human Nutritional Sciences, University of Manitoba, Winnipeg, MB R3T 2N2, Canada; 2 Department of Food Science, National Pingtung University of Science and Technology, Pingtung County 912301, Taiwan

**Keywords:** extrusion cooking, pulses, phenolic compounds, dietary fiber, texture, bowl life, color

## Abstract

Butterfly pea flower (BP) is a rich source of bioactive components and can potentially be utilized to produce appealing, wholesome foods. Antioxidant and dietary fiber-enriched breakfast cereals were produced by extrusion cooking using blends of BP and yellow pea flour (YP). BP was added to YP at 0%, 5% and 10% levels (*w/w*), respectively, and extruded at two temperature profiles with die temperatures of 130 and 150 °C. Incorporation of BP significantly (*p* < 0.05) improved the total phenolics content, antioxidant properties, and insoluble and total dietary fiber content of the extrudates, with 10% BP extrudates showing the highest values. At a die temperature of 150 °C, the extrudates had a higher expansion ratio, a lower dry hardness, and a higher dry crispiness as compared to those at 130 °C. The color of BP-incorporated extrudates was darker and bluer as compared to the no-BP extrudates. The 10% BP extrudates retained relatively more of their hardness, crispiness, and crunchiness after soaking, indicating a better bowl-life and, therefore, better suitability of this blend formula for breakfast cereal production. Overall, this research shows that healthier breakfast cereals with appealing color and relatively longer bowl-life can be produced using BP, making BP a potential novel ingredient for extrusion formulations.

## 1. Introduction

Extrusion processing is a highly efficient thermo-mechanical processing technology that is commonly employed to produce a wide range of food products such as puffed snacks, breakfast cereals, food ingredients, texturized vegetable proteins, and meat analogs [1,2]. The puffed snacks category is primarily composed of starch-rich expanded products because of starch’s ability to expand relatively better than proteinaceous raw materials, thereby providing better textural attributes and visual appeal [3,4]. However, the use of starch-rich raw materials (predominantly wheat, corn, potato, and rice) often makes the extrudates calorie-dense and nutrient-deficient [3]. Several efforts have been made in the past to increase the protein content of the extruded snacks by incorporating the pulse flours in extrusion formulations [5]. Among different pulse crops, yellow pea emerged to be one of the most promising raw materials to produce protein-rich puffed snacks [6,7,8]. In addition to being a rich source of proteins (18–28 g protein/100 g dry matter) [9], yellow pea also has a low-glycemic index, is a good source of dietary fiber (14–17 g dietary fiber/100 g dry matter) [10], and contains several bioactive compounds [11,12].

Bioactive compounds have been linked to several health benefits such as lower cardiovascular health risks, anti-cancer, and anti-diabetic properties [13]. One of the major categories of bioactive compounds is phenolics which, upon ingestion, offer several health benefits through their antioxidant and anti-inflammatory properties [14]. Butterfly pea flowers are a rich source of phenolic compounds (30 mg gallic acid equivalents/g dry matter) [15] and are often termed as a medicinal plant in many Asian countries [16,17,18]. The main phenolic compounds present in butterfly pea flowers are ternatin, kaempferol, quercetin, and myricetin [19]. The extracts of butterfly pea flowers have been reported to produce anti-allergic and anti-arthritic activities, hence highlighting their therapeutic capabilities [20,21]. The high anthocyanin content (9 mg ternatin B2 equivalents/g dry matter) [15] of butterfly pea flowers offers a wide array of opportunities to be used as a natural blue coloring agent in different food products [22]. Apart from bioactive components, edible flowers can also be consumed as a source of dietary fiber. It has been shown that the total dietary fiber content of edible flowers can reach up to 62 g/100 g flower (db) [23]. However, as per authors’ search on Web of Science, Scopus and PubMed databases using keywords “butterfly pea flower”, “Clitoria ternatea flower”, “soluble dietary fiber”, “insoluble dietary fiber” and “total dietary fiber”, no published research work was found on dietary fiber content of butterfly pea flowers. Since dietary fiber consumption offers several health benefits [24], butterfly pea flower as a novel ingredient to enhance the dietary fiber profile of foods warrants attention. Moreover, the effects of butterfly pea flower, more specifically its concentration, on the antioxidant properties of extruded foods such as breakfast cereals has not been investigated so far.

Despite being a rich source of several health-promoting components, the food uses of butterfly pea flowers are limited [17]. In a recently published study, butterfly pea flower was included at a 1% level in corn flour, sweet potato powder, and potato flake mixture to produce third-generation extruded snacks [25]. Considering their high content of bioactive compounds, butterfly pea flowers may potentially be incorporated at higher levels in extrusion formulations to produce healthier products. Potentially, due to the incompatibility of its particles with typical breakfast cereal ingredients (e.g., differences in flowability, surface charge, etc.), butterfly pea flower can potentially act as additional nucleation sites during extrusion cooking to help achieve a relatively more uniform microstructure in the end-product. However, the effects of butterfly pea concentration on extrudate properties should be carefully evaluated to minimize any adverse effects on their physical quality. The present study was carried out to produce nutritious puffed breakfast cereals using butterfly pea flower and yellow pea flour and to study the impact of butterfly pea flower concentration and extrusion temperature profile on the nutritional (i.e., antioxidant properties, dietary fiber content) and physical (i.e., expansion index, dry texture, wet texture) properties of extruded breakfast cereals.

## 2. Materials and Methods

### 2.1. Materials

Yellow pea flour (YP) with a protein (N × 6.25) content of 22.4 g protein/100 g flour (db), fat content of 0.6 g fat/100 g flour (db), and ash content of 2.6 g ash/100 g flour (db), respectively [8], was supplied by Ingredion Incorporated (Westchester, IL, USA). Butterfly pea flowers (BP) in dry form were supplied by Xi Fu Farm (Pingtung, Taiwan). The flowers were milled to pass through a 500 µm sieve using a centrifugal mill (ZM 200, Retsch, Haan, Germany). The flowers were milled in small batches to avoid over-heating of the centrifugal mill and ensure that the temperature remained <30 °C throughout the milling process. The proximate composition analysis of BP revealed 25.1 g/100 g (db) protein content, 2.1 g/100 g (db) fat content and 5.9 g/100 g (db) ash content, which were analyzed following AACC method 46-13 [26], Min and Ellefson [27] and Marshall [28], respectively. The total carbohydrate content, calculated by difference, was 74.4 g/100 g (db) for YP and 66.9 g/100 g (db) for BP. The dietary fiber content, i.e., total dietary fiber (TDF), soluble dietary fiber (SDF) and insoluble dietary fiber (IDF), of the raw flours and extrudates were measured by AACC method 32-07.01 [26]. The enzymes (α-amylase, protease and amyloglucosidase) used for dietary fiber analysis were purchased from Megazyme International Ltd. (Bray, Ireland).

Three blend formulations were prepared by mixing YP and BP using a Z-arm mixer before extrusion runs. BP was added to YP at concentrations of 0, 5, and 10% (*w/w*) to obtain the formulas 0% BP, 5% BP and 10% BP, respectively. These BP concentrations were selected on preliminary extrusion trials for optimal extrudate expansion.

### 2.2. Extrusion Processing

Extrusion experiments were performed on a lab-scale, co-rotating twin-screw extruder (MPF19, APV Baker Ltd., Peterborough, UK) equipped with a circular die of 2.3 mm diameter. The screw shafts were assembled according to Koksel and Masatcioglu [29], and screw speed was kept constant at 200 rpm. The feed rate and feed moisture content were also kept constant based on preliminary experiments at 2.86 kg/h (db) and 15 g water/100 g dry feed, respectively. Extrudates were produced using three different mixtures of YP and BP (0% BP, 5% BP, and 10% BP) at two different barrel temperature profiles, i.e., 60–80–90–110–130 °C and 60–80–100–130–150 °C (five temperature-controlled zones from the feeder to the die end of the extruder barrel). At all processing conditions, extrudates were collected in duplicates after attaining steady state conditions and cooled down to room temperatures. The extrudates were then oven dried (Heratherm OGS100, Thermo Fisher Scientific, Dreieich, Germany) overnight at 50 °C to reduce their moisture content below 10% for long term storage at ambient temperature. After drying, extrudates were placed in zipped plastic bags to avoid any moisture change. A small part of the extrudates was milled to pass through a 250 µm sieve using a centrifugal mill (ZM 200, Retsch, Haan, Germany) for further analyses.

### 2.3. Total Phenolic Compounds (TPC) and Antioxidant Properties

#### 2.3.1. Sample Extraction

Ultrasonic-assisted aqueous extraction was used for preparing the samples for TPC and antioxidant properties analyses following Altemimi et al. [30] with some modifications. In brief, 10 mL extraction mixture was prepared using 0.1 g of milled raw flour or extrudates and appropriate amount of Milli Q water. This mixture was mixed properly using a vortex mixer (G-560, Scientific Industries Inc., Bohemia, NY, USA) and extracted using an ultrasonic bath (Retsch, UR 3, Haan, Germany) for 30 min. The mixture was then centrifuged (Sorvall RC6 plus, Thermo Fisher Scientific, Asheville, NC, USA) for 20 min at 5150× *g*. The extracted supernatant (extract) was then filtered (Whatman no. 2) and stored for further analyses.

#### 2.3.2. TPC

The TPC content of the raw flours and extrudates was determined according to Oktay et al. [31] with minor modifications. Briefly, 300 µL of the raw flour (or extrudate) extract was mixed with 300 µL Folin–Ciocalteu reagent in 1.5 mL Eppendorf tubes. After keeping this mixture at room temperature for 5 min, 600 µL of 20% Na_2_CO_3_ was added, vortexed, and kept at a dark place for 30 min, followed by centrifugation (Micro-12, Fisher Scientific, Waltham, MA, USA) for 20 min at 7200× *g*. The absorbance of the supernatant was measured at 765 nm using a UV spectrophotometer (Ultrospec 1100 Pro, Biochrom, Cambridge, UK). A standard gallic acid curve was prepared, and the results were calculated as gallic acid equivalents (GAE) from the standard curve. All measurements were performed in triplicates.

#### 2.3.3. DPPH (1,1-Diphenyl-2-picryl-hydrazil) Scavenging Ability

The DPPH scavenging ability of the extrudates was measured (in triplicates) according to the method of Oktay, Gülçin and Küfrevioğlu [31]. Using 1.5 mL Eppendorf tubes, 500 µL of 0.2 mM DPPH solution was added to 500 µL of either extrudate extract or Milli Q water (for blank). After proper mixing, the tubes were centrifuged at 7200× *g* for 10 min and the absorbance was measured at 517 nm. The DPPH scavenging ability was calculated using the equation below:

DPPH scavenging ability (%) = (1 − (Absorbance_extract)/(Absorbance_blank)) × 100(1)

#### 2.3.4. Reducing Power

The reducing power of the extrudates was measured (in triplicates) following Oktay, Gülçin and Küfrevioğlu [31]. For this analysis, 300 µL of phosphate-buffered solution and 300 µL of 1% red prussiate (K_3_Fe(CN)_6_) solution were added to extrudate extract or Milli Q water (blank) in 15 mL falcon tubes. The tubes were then vortexed and kept in a water bath at 50 °C for 20 min followed by the addition of 300 µL trichloroacetic acid (10%), 1.2 mL distilled water, and 1.2 mL ferric chloride (0.1%) solution. The tubes were then kept in dark for 10 min and the absorbance was measured at 700 nm. The reducing power was calculated by subtracting the absorbance value of the blank from that of the extract.

### 2.4. Physical Properties

#### 2.4.1. Expansion Index

Extrudates of approximately 10 cm length were randomly selected (in triplicates) and their diameter was measured using a caliper of 0.01 mm accuracy along the extrudate length at 2, 4, 6, and 8 cm. From the average of these measurements, the expansion index was calculated by taking the ratio of diameter of extrudate to the diameter of die used (i.e., 2.3 mm).

#### 2.4.2. Texture Analysis

Textural properties of the extrudates were measured using a texture analyzer (TA-XT-plus, Stable Micro Systems, Gudalming, UK) following Koksel and Masatcioglu [29]. From each processing condition, randomly selected extrudates were cut into 4 cm long pieces and dried overnight at 50 °C for moisture equilibration. Using a 1 mm thick Warner-Bratzler shear probe blade and 5 kg load cell, 15 randomly selected extrudate pieces (length 4 cm) were individually cut to 50% of their original thickness at a shear angle of 90° with a downward and upward probe travel speed of 2 mms^−1^ and 10 mms^−1^, respectively. Different textural parameters, i.e., hardness (peak force, N), crispiness (number of positive peaks) and crunchiness (linear distance of the curve, N s) were obtained from the force vs. time plot. For wet texture (i.e., bowl life), extrudates were prepared according to Luo and Koksel [7] and placed in cold water for 3 min followed by draining the water for 10 s. The wet textural attributes were measured using the same steps as described above for dry texture.

#### 2.4.3. Color

The color attributes of the raw flours and their extruded counterparts was measured in triplicates using a color spectrophotometer (CM-3500d, Minolta, Osaka, Japan). The raw flours and extrudates were first milled and then filled into a cylindrical glass measurement container, and the values for different color parameters, i.e., L* (higher values depict lighter color), a* (positive a* value depicts redness and negative a* value depicts greenness), b* (positive b* value depicts yellowness and negative b* value depicts blueness) were obtained using Spectra Magic (Version 3.60) software. ΔE, i.e., total color change between extrudates and their raw counterparts, was calculated using the equation given by [32].

### 2.5. Statistical Analysis

Statistical differences (*p* < 0.05) among extrusion treatments were determined by one-way ANOVA using SAS software (Version 9.2, SAS Institute Inc., Cary, NC, USA).

## 3. Results and Discussion

### 3.1. TPC and Antioxidant Properties

The TPC content of raw YP was 3.0 ± 0.1 mg GAE/g (db) which is in line with the previously reported range (2.2–9.1 mg GAE/g) for split and whole yellow peas of different particle sizes [33]. As expected, the raw BP contained a much higher amount of TPC than YP with a value of 49.2 ± 0.8 mg GAE/g (db), again in agreement with the TPC range reported for BP in the literature [34,35]. It has also been reported that the TPC content of the raw BP greatly depends on variety [35], extraction solvent used, e.g., water or methanol [36], and extraction method, e.g., ultrasound or water bath [34].

**Figure 1:**
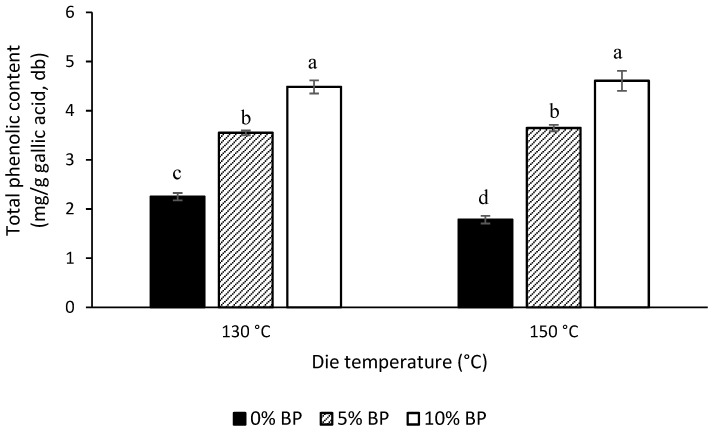
Total phenolic content (mean ± standard deviation) of extrudates as a function of extrusion die temperature and butterfly pea flower (BP) concentration. Bars assigned with same letters are not significantly (*p* < 0.05) different (*n* = 2 for extrusion, *n* = 3 for total phenolic content).

The TPC content of extrudates as a function of extrusion die temperature and feed formulation are provided in Figure 1. Extrusion processing resulted in approximately 25 to 41% reduction in the TPC content of YP. This decrease was expected due to the high temperature applied during extrusion processing. Extrusion processing was shown to cause 10 and 70% reduction in the TPC values of navy bean and red bean flours, respectively [37]. High temperature may cause degradation of temperature sensitive phenolic compounds, mainly through their decarboxylation [38,39]. Addition of BP in blend formulation resulted in a significant (*p* < 0.05) increase in the TPC content of the extrudates, with 10% BP showing the highest values followed by 5% BP and 0% BP (Figure 1). This increase in TPC shows the excellent potential of BP to be used in the manufacture of healthy puffed snacks and breakfast cereals. For 0% BP, the increase in die temperature caused a significant decrease in TPC, while the TPC values were independent of die temperatures studied for BP enriched extrudates. Harsh thermal conditions can affect the TPC in both positive and negative ways. On one hand, an increase in extrusion temperature can promote decarboxylation to reduce the TPC [40], in line with the trends observed in the present study for 0% BP extrudates. While on the other hand, harsh thermal conditions can favor the extraction of some of the polyphenolic compounds from the cell wall matrix through hydrolysis of larger polyphenols [41,42]. Accordingly, the stable TPC content observed in BP enriched extrudates may be a result of the cumulative effects of these two competing factors.

**Figure 2:**
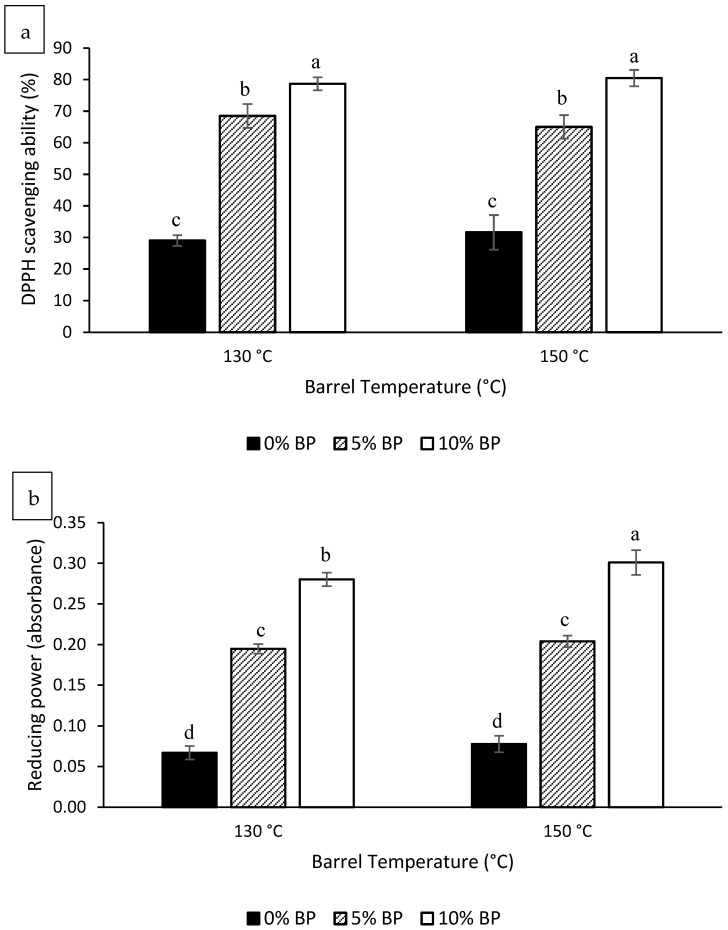
DPPH (1,1-diphenly-2-picryl-hydrazil) scavenging ability (**a**) and reducing power (**b**) (mean ± standard deviation) of extrudates as a function of extrusion die temperature and butterfly pea flower (BP) concentration. Bars assigned with same letters are not significantly (*p* < 0.05) different (*n* = 2 for extrusion, *n* = 3 for total DPPH scavenging ability and reducing power).

The results for the DPPH scavenging ability of the extrudates are provided in Figure 2a. The DPPH scavenging ability of the extrudates significantly increased as a function of increasing BP concentration. Similar trends were also observed for the reducing power of the extrudates (Figure 2b). This increase in DPPH scavenging ability and reducing power after BP addition reflects the strong antioxidant properties of the phenolic compounds present in BP. No significant change in antioxidant properties of the extrudates was observed when the extrusion die temperature was increased from 130 °C to 150 °C, expect for reducing power of 10% BP extrudates, which showed a significant (*p* < 0.05) increase with an increase in die temperature.

### 3.2. Dietary Fiber Content

Insoluble dietary fiber (IDF), soluble dietary fiber (SDF), and total dietary fiber (TDF) contents of raw YP, raw BP, and the extrudates are presented in Table 1. The IDF, SDF, and TDF contents of raw YP were comparable with the values reported in the literature for yellow peas [10,43]. Compared to raw YP, the raw BP had significantly higher IDF, SDF, and TDF (Table 1). The theoretical IDF, SDF, and TDF values of raw blends as calculated from the IDF, SDF, and TDF values of raw YP and raw BP were 14.20, 2.02, and 16.22 g/100 g (db), respectively, for raw 5% BP and 14.82, 2.15, and 16.97 g/100 g (db), respectively, for raw 10% BP. Accordingly, the addition of BP to YP at the level of 5% and 10% (*w/w*) slightly increased the IDF, SDF, and TDF of the feed formulations.

Compared to the raw blends, the IDF and TDF contents generally decreased for extrudates (Table 1), which are in agreement with previous findings for extrusion of pea flour [44]. Similar findings were also reported for extrusion of lentils [42] and wheat bran [45,46,47]. For instance, Frias, Giacomino, Peñas, Pellegrino, Ferreyra, Apro, Carrión, and Vidal-Valverde [44] reported a decrease in TDF content of pea flour from 18.3 to 15.3 g/100 g (db) after extrusion processing at 129 °C die temperature. The decrease in IDF and TDF might be attributed to the degradation of some dietary fiber components to low molecular weight fractions upon exposure to high temperature and high shear conditions during extrusion cooking [45,47]. Accordingly, the alcohol precipitation method which was used in the present study might not fully recover these newly formed low molecular weight dietary fiber fractions [48]. Despite significant reduction in IDF and TDF of the extrudates, comparison of the SDF contents of raw YP and extrudates showed that SDF content did not significantly (*p* < 0.05) change as a result of extrusion (Table 1). Although some extrusion studies showed an increase in SDF due to extrusion processing [42,49], the effects of extrusion on fiber solubility highly depends on the extrusion conditions and the feed characteristics [47,50]. For instance, Berrios, Morales, Cámara, and Sánchez-Mata [50] reported an increase in SDF content of peas after extrusion processing at 17% moisture content and 160 °C die temperature, while they reported no significant changes in SDF content of lentils after extrusion. The differences in raw material characteristics and extrusion conditions might be responsible for the observed differences in the effects of extrusion processing on SDF content. In terms of die temperature, no significant effects (*p* < 0.5) on IDF, SDF, and TDF were observed when the die temperature was increased from 130 °C to 150 °C except for 10% BP extrudates for which an increase in die temperature significantly (*p* < 0.05) improved the IDF and TDF content (Table 1). Overall, with the addition of BP to YP at 10%, extrudates with relatively higher TDF content (14.8–16.4 g/100 g, db) were obtained when compared to the previously published studies on fiber enriched puffed extruded products, for example, extrusion of barley and carrot pomace blends (13 g/100 g, db) [51], and maize, flaxseed and amaranth blends (13.5 g/100 g, db) [52].

A comparison of extrudates obtained from different feed formulations showed that BP addition significantly (*p* < 0.05) improved the IDF and TDF content of extrudates, with 10% BP having the highest IDF and TDF content (Table 1). The SDF content of extrudates was not significantly (*p* < 0.05) affected by the incorporation of BP at the level of 5% and 10%. Considering the important role of dietary fibers as essential nutritional components in human health such as their role in the reduction in risks of cardiovascular diseases and type II diabetes [24], the addition of 10% BP into the extrusion formula can provide consumers with healthier options in breakfast cereals. Future studies can focus on incorporation of BP in a variety of food products for possible nutrient content claims.

### 3.3. Physical Properties

#### 3.3.1. Expansion Index

**Figure 3:**
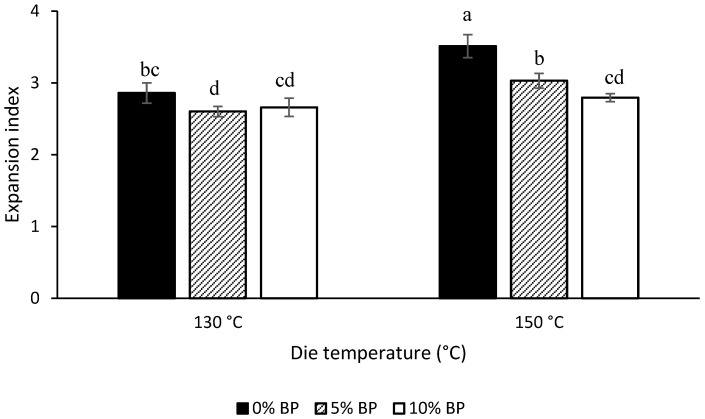
Expansion index (mean ± standard deviation) of extrudates as a function of extrusion die temperature and butterfly pea flower (BP) concentration. Bars assigned with same letters are not significantly (*p* < 0.05) different (*n* = 2 for extrusion, *n* = 3 for expansion index).

The effects of extrusion die temperature and feed formulation on extrudate expansion index are presented in Figure 3. Overall, all extrusion treatments produced well-expanded extrudates, with expansion index values ranging from 2.6 to 3.5. Comparing with literature works on producing high-protein extruded snacks [32,37,53], the protein content (22.4–22.7 g protein/100 g, db) and expansion index (2.6–3.5) of extrudates produced in the present study were relatively high. For instance, Muñoz-Pabon, Parra-Polanco, Roa-Acosta, Hoyos-Concha, and Bravo-Gomez [53] reported an expansion index in the range of 1.2–2.3 for multi-cereal blends having protein content between 7.0 and 16.2 g protein/100 g, db, and Anton, Fulcher, and Arntfield [37] produced expanded puffed snacks from common bean–corn starch blends having protein contents between 3.0 and 10.1 g protein/100 g, db and expansion indices between 1.7 and 2.2. In general, an increase in expansion index was observed with increasing die temperature from 130 °C to 150 °C. Increased expansion at higher die temperatures might be attributed to the higher temperature differential when the melt exits the die, which favors relatively fast water evaporation and consequently improves overall extrudate expansion [54]. Moreover, high temperatures also result in a higher degree of starch gelatinization in the extruder barrel which may also positively affect the expansion ratio [55]. However, higher temperatures have also been generally associated with lower die pressures due to decreased melt viscosity inside the barrel [56,57], which may negatively impact the extrudate expansion ratio. The raw material composition is highly important to dictate the effects of temperature on the final product characteristics. For instance, in the current study, the total carbohydrate content of BP is approximately 7.5% lower while its total dietary fiber content is significantly (*p* < 0.05) higher than that of YP (Table 1). Since dietary fiber is a subset of total carbohydrates content, these numbers suggest a relatively higher starch content in the YP compared to that in the BP. Accordingly, the decreased starch content in the extrusion feed formulation after BP addition may be responsible for the effects of increasing temperature not being observed for the 10% BP extrudates, unlike the effects observed for 0% BP and 5% BP.

In terms of BP addition, expansion index showed a decreasing trend with the incorporation of BP. This trend was more prevalent and statistically significant (*p* < 0.05) at 150 °C die temperature. The decrease in expansion with the incorporation of BP may be attributed to the higher dietary fiber content of BP (Table 1). A decrease in expansion index of extrudates obtained from higher dietary fiber containing blends was also reported by Li, Guillermic, Nadimi, Paliwal, and Koksel [32] at similar extrusion processing conditions. As the dietary fiber content of BP incorporated extrudates increase at the expense of their starch content and since expansion index primarily depends on the starch properties [58], this starch dilution effect of dietary fiber may be responsible for the observed decrease in the expansion of 5% and 10% BP extrudates. Moreover, dietary fiber (especially its insoluble fractions) can impact the water distribution in the melt and consequently its viscosity inside the barrel due to its relatively more hydrophobic nature compared to starch [48]. While low levels of dietary fiber do not normally produce a negative impact on extrudate expansion, high dietary fiber levels might impair the expansion by hindering bubble formation due to disruption of the continuous starch phase [48,58].

#### 3.3.2. Texture

The effects of extrusion barrel temperature and feed formulation on dry and wet (bowl-life) texture of the extrudates are presented in Table 2. An increase in die temperature from 130 °C to 150 °C caused a significant (*p* < 0.05) decrease in hardness in both dry and wet conditions. This is in line with the literature for the extrusion of barley–tomato pomace blends [59] and wheat flour [60] at similar die temperatures. The lower hardness for extrudates obtained at 150 °C can be attributed to their higher expansion (Figure 3) as more expanded structures generally result in softer extrudates [56,60]. Similarly, a negative correlation between expansion index and extrudate hardness was previously reported for pea flour extrudates [29]. In dry conditions, the BP addition did not cause any significant changes in hardness values at both extrusion die temperatures. However, at 150 °C die temperature, the wet hardness for 10% BP was significantly higher as compared to 0% BP extrudates, meaning that 10% BP containing formula did not become softer as fast as the no BP containing formula, making it more suitable for applications such as breakfast cereals. Overall, the extrudates hardness was generally lower in wet conditions than in dry conditions, which is expected and in line with the results reported by Luo and Koksel [7].

Contrary to hardness, the crispiness of the extrudates in the dry form generally increased when the die temperature was increased from 130 °C to 150 °C. Crispiness typically shows a positive relationship with extrudate expansion [7]; hence, these findings are consistent with the previously observed trends. However, after immersion in water, the extrudates produced at the higher die temperature significantly lost their crispiness. Water generally induces a plasticizing effect on the breakfast cereals, making them soggy and less acceptable to consumers. Since higher crispiness is one of the critical attributes for dictating consumer’s acceptability [61], extrudates which retain their structures after soaking are generally considered more desirable for breakfast cereals applications. For the extrudates obtained at 150 °C die temperature, only 10% BP extrudates retained their crispiness values in wet conditions, inline with the findings for hardness values, making them more suitable for breakfast cereals applications.

The results for crunchiness showed no significant changes as a function of extrusion die temperature or feed formulation in their dry form. For bowl life (i.e., wet texture), a general decrease in crunchiness values was observed when the die temperature was increased. No significant change in the crunchiness values was observed between dry and wet conditions for extrudates produced at 130 °C. However, for extrudates produced at 150 °C, the crunchiness of 0% BP and 5% BP extrudates was significantly reduced in wet conditions as compared to their dry counterparts. Like crispiness, the crunchiness values for 10% BP were relatively more stable in their wet form. Considering the importance of these textural attributes for the acceptability of breakfast cereals, the addition of BP was found to be beneficial in producing healthy and more appealing breakfast cereals.

#### 3.3.3. Color Analysis

The color parameters of raw blends and extrudates as a function of extrusion die temperature and BP concentration are provided in Table 3. The raw blends with 5% and 10% BP concentration were darker (lower L*), greener (negative a*), and bluer (negative b*) in color in comparison with raw YP. These differences in color attributes are due to the presence of anthocyanins in BP which provide them a dark and blue color [62]. After extrusion processing, the L* value of YP decreased from 89.46 (raw) to 83.15 (extruded) at die temperature of 130 °C. Further increase in die temperature to 150 °C reduced the L* value further to 80.72. This decreasing trend in L* after extrusion processing is widely reported in the literature which most likely happens because of a reaction between amino acids and sugars (commonly known as the Maillard reaction) at high temperatures [63,64,65].

As expected, due to natural color of butterfly pea flowers, the lowest L* (i.e., darkest color) values were observed for 10% BP extrudates. Similarly, a* and b* values also significantly decreased for BP enriched extrudates as compared to 0% BP. The total color change, i.e., ΔE was significantly (*p* < 0.05) high for BP enriched blends (Table 3). The degradation of some color pigments at high temperatures may also be a possible reason for the color change after extrusion processing [59,64]. Since BP is a rich source of color pigments such as anthocyanins [18], the higher differences in the total color change (i.e., ΔE) may be due to degradation of anthocyanins at high temperatures that were used during the extrusion process.

## 4. Conclusions

Extruded breakfast cereals were prepared from unique blends of yellow pea flour (high in protein) and butterfly pea flower (high in antioxidants and dietary fiber) at two extrusion die temperatures, i.e., 130 and 150 °C. The incorporation of butterfly pea flower (BP) at the level of 10% (*w/w*) to yellow pea flour resulted in a significant increase in the total phenolic compounds, antioxidant properties, as well as the insoluble and total dietary fiber content of the extrudates when compared to extrudates produced with no BP addition. These results indicate that BP-enriched extruded breakfast cereals may provide several health benefits. The physical and textural characteristics of the extrudates were significantly improved with an increase in die temperature. Although the expansion index of 10% BP extrudates was lower compared to 0% BP, their relatively higher structural stability after soaking in water may provide benefits in the manufacture of breakfast cereals. The present research is the first to explore the possibilities of using BP at levels that have not been studied before for pulse-based extruded puffed snacks and breakfast cereals. More research is warranted to optimize the extrusion parameters such as temperature, feed moisture, and screw speed to further improve the physical attributes of BP-enriched snacks and for possible nutrient content claims for dietary fiber content. Future work will also explore the extent of the impact of BP addition on the protein nutritional quality and vitamin profile of extruded breakfast cereals.

## Figures and Tables

**Table 1:** Insoluble dietary fiber (IDF), soluble dietary fiber (SDF), and total dietary fiber (TDF) (mean ± standard deviation) of raw materials and extrudates as a function of extrusion die temperature and butterfly pea flower (BP) concentration. Values in the same column followed with same letters are not significantly (*p* < 0.05) different (*n* = 2 for dietary fiber measurements).

Blend	Die Temperature (°C)	IDF (g/100 g, db)	SDF (g/100 g, db)	TDF (g/100 g, db)
Raw yellow pea	-	13.59 ± 0.14 ^b,c^	1.89 ± 0.02 ^b^	15.48 ± 0.12 ^b,c^
Raw butterfly pea flower	-	25.86 ± 0.25 ^a^	4.54 ± 0.21 ^a^	29.37 ± 0.95 ^a^
0% BP	130	10.10 ± 0.21 ^f^	1.45 ± 0.03 ^b,c^	11.56 ± 0.21 ^f^
	150	10.02 ± 0.46 ^f^	1.99 ± 0.16 ^b,c^	12.01 ± 0.38 ^f^
5% BP	130	11.20 ± 0.27 ^e,f^	2.08 ± 0.09 ^b^	13.31 ± 0.21 ^e^
	150	11.79 ± 0.23 ^d,e^	2.04 ± 0.04 ^b,c^	13.83 ± 0.25 ^d,e^
10% BP	130	13.08 ± 0.40 ^c,d^	1.67 ± 0.17 ^b,c^	14.78 ± 0.28 ^c,d^
	150	15.12 ± 0.08 ^b^	1.32 ± 0.16 ^c^	16.44 ± 0.11 ^b^

**Table 2:** Textural properties (mean ± standard deviation) of extrudates as a function of extrusion die temperature and butterfly pea flower (BP) concentration. Values in the same column followed with same letters are not significantly different (*n* = 2 for extrusion runs and *n* = 3 for textural attributes).

Blend	Die Temperature (°C)	Hardness (N)	Crunchiness (Ns)	Crispiness
		Dry texture		
0% BP	130	11.25 ± 0.66 ^a,b^	40.83 ± 5.07 ^a^	6.10 ± 1.37 ^b^
	150	6.78 ± 1.01 ^e,f^	33.88 ± 2.72 ^a,b^	10.63 ± 2.10 ^a^
5% BP	130	11.71 ± 1.64 ^a^	40.20 ± 6.88 ^a^	6.00 ± 1.04 ^b^
	150	7.58 ± 1.54 ^d,e,f^	36.64 ± 5.67 ^a,b^	9.73 ± 1.23 ^a^
10% BP	130	10.10 ± 0.68 ^a,b,c^	38.06 ± 4.79 ^a,b^	6.23 ± 1.49 ^b^
	150	7.44 ± 1.30 ^d,e,f^	29.99 ± 4.59 ^b,c,d^	7.23 ± 1.46 ^b^
		Wet texture (bowl-life)		
0% BP	130	9.95 ± 1.23 ^a,b,c^	33.23 ± 3.18 ^a,b^	6.57 ± 1.06 ^b^
	150	3.34 ± 1.17 ^g^	13.05 ± 4.61 ^e^	6.27 ± 1.29 ^b^
5% BP	130	9.11 ± 1.40 ^b,c,d^	34.23 ± 4.12 ^a,b^	6.80 ± 0.61 ^b^
	150	5.49 ± 0.82 ^f,g^	20.92 ± 4.73 ^d,e^	6.76 ± 0.96 ^b^
10% BP	130	8.58 ± 0.86 ^c,d,e^	32.13 ± 6.75 ^a,b,c^	6.67 ± 1.06 ^b^
	150	6.19 ± 0.53 ^f^	23.41 ± 3.19 ^c,d^	7.17 ± 0.53 ^b^

**Table 3:** Color parameters (mean ± standard deviation) of extrudates as a function of extrusion die temperature and butterfly pea flower (BP) concentration. Values in the same column followed by same letters are not significantly (*p* < 0.05) different (*n* = 2 for extrusion and *n* = 3 for color measurements).

Blend	Die Temperature (°C)	L*	a*	b*	ΔE
Raw 0% BP	-	89.46 ± 0.06 ^a^	0.73 ± 0.03 ^b^	16.05 ± 0.13 ^b^	-
0% BP	130	83.15 ± 0.25 ^c^	2.04 ± 0.08 ^a^	22.91 ± 0.55 ^a^	9.42 ± 0.56 ^e^
	150	80.72 ± 0.33 ^d^	1.73 ± 0.36 ^a^	22.25 ± 0.77 ^a^	10.79 ± 0.26 ^d^
Raw 5% BP	-	84.30 ± 0.27 ^b^	−1.13 ± 0.07 ^c^	11.15 ± 0.27 ^c^	-
5%BP	130	50.86 ± 0.71 ^e^	−4.32 ± 0.08 ^g^	−8.62 ± 0.13 ^g^	38.98 ± 0.65 ^b^
	150	49.68 ± 0.41 ^f^	−4.59 ± 0.08 ^g^	−4.31 ± 0.81 ^e^	38.08 ± 0.61 ^c^
Raw 10% BP	-	80.15 ± 0.08 ^d^	−1.71 ± 0.04 ^d^	9.33 ± 0.08 ^d^	-
10% BP	130	43.39 ± 0.43 ^g^	−2.80 ± 0.08 ^e^	−11.32 ± 0.09 ^h^	42.17 ± 0.36 ^a^
	150	41.84 ± 0.28 ^h^	−3.35 ± 0.10 ^f^	−6.74 ± 0.28 ^f^	41.58 ± 0.23 ^a^

## Data Availability

The data are available on request from the corresponding author.
